# AC-CUTE: An Open-Label Study to Evaluate Progression of Structural Joint Damage and Inflammation in Subjects with Moderate to Severe Rheumatoid Arthritis

**DOI:** 10.1155/2018/8721753

**Published:** 2018-04-12

**Authors:** Paul Bird, Charles Peterfy, Peter Countryman, Hedley Griffiths, Rina Barrett, Peter Youssef, Fredrick Joshua, Stephen Hall

**Affiliations:** ^1^University of New South Wales, Sydney, NSW, Australia; ^2^Combined Rheumatology Practice, Kogarah, NSW, Australia; ^3^Spire Sciences, Inc., Boca Raton, FL, USA; ^4^Barwon Rheumatology Service, Geelong, VIC, Australia; ^5^Roche Products Pty, Limited (Australia), Sydney, NSW, Australia; ^6^Royal Prince Alfred Hospital, Camperdown, NSW, Australia; ^7^Cabrini Medical Centre, Malvern, VIC, Australia

## Abstract

**Aim:**

Examine the efficacy of once-weekly subcutaneous tocilizumab (SC-TCZ) on joint damage at 24 weeks based on radiography of the hands and feet and magnetic resonance imaging (MRI) of the hand in subjects with moderate to severe rheumatoid arthritis (RA).

**Methods:**

In this Australian open-label, multicentre, prospective, single-arm study, subjects received 162 mg SC-TCZ weekly. Primary endpoint was change in radiographic Genant-modified Total Sharp Score (TSS) between baseline and Week 24. Secondary endpoints included change from baseline to Week 24 in RA MRI scoring (RAMRIS) of erosions, synovitis, and osteitis and Cartilage Loss Score (CARLOS) in the dominant hand and disease activity score 28 (DAS28).

**Results:**

52 subjects were enrolled (80% female, mean (SD) age 57  (12) years). Radiography showed mild but not significant progression of joint damage (mean (SD) change in TSS 0.46 (1.29)). Synovitis reduced significantly on MRI; however, osteitis, erosion, and cartilage loss did not change significantly. DAS28 improved significantly by Week 24; 78% of subjects achieved DAS28 remission. SC-TCZ was generally well tolerated.

**Conclusion:**

Synovitis and DAS28 decreased significantly; however, no significant change in osteitis or joint damage was observed at Week 24.

**Trial registration:**

This trial is registered with Clinicaltrials.gov registration number NCT01951170 (ML28703).

## 1. Introduction

Imaging assessment of structural damage in subjects with rheumatoid arthritis (RA) has been traditionally centred around conventional radiography (X-ray). However, X-ray cannot provide information regarding inflammatory changes in synovium, bone, or tendon sheaths and is relatively insensitive for bone erosion. Further, it cannot define articular cartilage loss directly, and thus cartilage loss can only be surmised by narrowing of the lucent joint space. Accordingly, physicians and investigators are turning increasingly to magnetic resonance imaging (MRI) for earlier detection of bone erosion, osteitis, and synovitis and to evaluate the integrity of articular cartilage, tendons, ligaments, and other structures important to joint function.

Tocilizumab administered intravenously (IV-TCZ) has previously been shown to inhibit progression of X-ray joint damage within 24 weeks in two phase 3 studies of subjects with moderate to severe RA (LITHE and ACT-RAY) [1, 2]. In an MRI substudy of ACT-RAY, IV-TCZ was shown to decrease synovitis and osteitis within 2 weeks and inhibit bone erosion within 12 weeks [3].

Noninferiority of a subcutaneous formulation of TCZ (SC-TCZ, 162 mg weekly) compared to IV-TCZ 8 mg/kg every 4 weeks has been demonstrated in terms of the proportion of subjects in each group achieving an American College of Rheumatology (ACR) 20 response at Week 24 [4].

The AC-CUTE study examined the effects of SC-TCZ 162 mg weekly for 24 weeks on X-ray and MRI joint damage in subjects with moderate to severe active RA, who had inadequate response to MTX or were considered unsuitable for MTX treatment due to intolerance or other reasons or who had experienced an inadequate response to a single antitumour necrosis factor (TNF) therapy. At the time of study initiation, MRI trials demonstrated erosion progression within only 12 weeks [3]. Accordingly, most recent trials have limited MRI follow-up to 12 or 24 weeks. In this study, follow-up was similarly limited to only 24 weeks because of this as well as budgetary reasons. The association between osteitis and synovitis at baseline and the progression of erosion on follow-up at the level of individual bones and joints was also examined, with the aim of providing data to help enrich future clinical trial cohorts with subjects more likely to progress structurally.

## 2. Patients and Methods

### 2.1. Study Design

AC-CUTE (NCT01951170) was a phase IIIb, open-label, multicentre, prospective, single-arm study to evaluate the efficacy of once-weekly SC-TCZ in preventing progression of joint damage in RA. This Australian substudy is part of an umbrella project consisting of several independent studies with similar designs conducted in various countries [5]; safety and efficacy data will be pooled for a global analysis, with the primary objective of assessing safety of SC-TCZ.

### 2.2. Subjects

Subjects were ≥18 years old and had moderate to severe active RA according to the revised 1987 ACR criteria [6] or 2010 EULAR/ACR criteria [7], CRP ≥ 1 mg/dL or ESR ≥ 28 mm/hr, no previous exposure to TCZ, and evidence of ≥1 erosions attributable to RA on X-ray of both hands and feet or MRI of the dominant hand and wrist, at screening.

Exclusion criteria included serious comorbidities, major surgery within 8 weeks prior to screening or planned to occur within 6 months of baseline, significant systemic RA involvement, functional class-IV RA, other inflammatory joint diseases, or current/recurrent infections. Prohibited therapies included previous cell-depleting therapies, alkylating agents, or ≥2 anti-TNF therapies or any other biologic agent; or treatment with IV-gamma globulin or plasmapheresis within 6 months of baseline; or intra-articular or parenteral corticosteroids within 4 weeks prior to baseline.

Participating subjects provided written informed consent. The study was conducted in accordance with local guidelines and in line with the principles of the Declaration of Helsinki and Good Clinical Practice Guidelines. Ethics approval was obtained from Bellberry Human Research Ethics Committee for 7 sites (2013-05-250); the remaining 3 sites obtained approvals from Royal Prince Alfred Hospital Ethics Review Committee (HREC/13/RPAH/280), Tasmanian Health and Medical Research Human Ethics Committee (H0013269), and Royal Adelaide Hospital Research Ethics Committee (130629).

### 2.3. Study Treatments

SC-TCZ given as 0.9 mL of a 180 mg/mL solution (i.e., 162 mg fixed dose irrespective of body weight), in a single-use, prefilled syringe, was administered weekly, for 24 weeks. Allocation of subjects to SC-TCZ monotherapy or SC-TCZ in combination with MTX or disease-modifying antirheumatic drug(s) (DMARD(s)) was at the discretion of the treating physician.

Subjects or their caregivers were trained by a health care professional (HCP) to perform the injection. The first SC-TCZ injection was administered under supervision. Once the HCP was satisfied the injection could be given competently, subjects could choose to administer SC-TCZ at home between assessment visits.

### 2.4. Assessments and Procedures

Assessments were performed at Weeks 1 (baseline), 2, 4, 8, 12, 16, 20, and 24 during the treatment period and at study completion. X-ray was performed at screening (within 4 weeks prior to baseline) and at Week 24. MRI was performed at baseline and Week 24.

Assessments included patient reported outcome (PRO), including the patient's global assessment of disease activity Visual Analogue Scale (VAS), pain VAS, Health Assessment Questionnaire-Disability Index (HAQ-DI), and subject compliance; laboratory samples (taken after PRO assessments but prior to study drug administration); efficacy assessments including joint counts, physician's global assessment of disease activity VAS, change in DAS28 (ESR CRP), ACR response scores, EULAR response criteria, SDAI/CDAI, documentation of nonbiologic DMARD, and corticosteroid dose reductions and/or discontinuations. Safety was assessed from reports of adverse events (AEs), vital signs, physical examination, concomitant medications, and review of laboratory data at least from the previous visit before the next dose of TCZ was dispensed. A safety follow-up visit occurred 8 weeks after final treatment.

Immunogenicity (anti-TCZ antibodies, TCZ levels, and sIL-6R) was assessed at baseline and at Weeks 12 and 24 and 8 weeks after last dose as well as withdrawal due to anaphylaxis or hypersensitivity reactions.

X-ray of both hands and feet and MRI of the dominant hand were performed using specialised positioning frames (X-Frame™ for X-ray and M-Frame™ for MRI, Spire Sciences, Inc.) to ensure reproducible imaging. All subjects had 1.5 T MRI of the dominant hand and wrist using a knee coil with the subject prone and the arm over the head. Pulse sequences included coronal and axial short-tau inversion recovery (STIR) and coronal fat-saturated T1-weighted three-dimensional gradient echo (3D-GRE) scans. Coronal and axial slices were each aligned using two orthogonal localizer scans to improve reproducibility between baseline and Week 24 scans. Gadolinium-containing contrast was not used. All images were checked centrally for graphic quality and compliance with the study protocol before inclusion in the analysis. Two central radiologists independently reviewed all images, blinded to subjects' treatments, each other's scores, and the temporal order of the scans. The top 10% of discrepant change scores between the two radiologists were adjudicated by consensus review to identify potential input errors. Otherwise, the scores of the two readers were averaged.

X-ray images were scored for bone erosion, joint-space narrowing (JSN), and Total Sharp Score (TSS) using the Genant-modified Sharp method [8, 9]. MR images were scored for bone erosion, osteitis, and synovitis using the Outcome Measures in Rheumatology (OMERACT) RA MRI Score (RAMRIS) [10] and for articular cartilage loss using the Cartilage Loss Score (CARLOS) method [11].

### 2.5. Statistical Considerations and Analytical Plans

The primary efficacy analysis was descriptive and assessed the change in progressive joint destruction by X-ray between screening and Week 24. The primary endpoint was the absolute change in TSS between baseline and Week 24. The Wilcoxon rank-sum test was used to test the null hypothesis of no change from baseline at Week 24 in the total score for each MRI or radiographic feature. In addition, proportions of subjects with changes in total feature scores ≥ Smallest Detectable Change (SDC) (SDC = 1.96 × SD_difference_ in change score between readers/(√2√*k*), where* k* = number of readers) [12] at Week 24 were determined.

Imputation was performed for missing scores at the joint/bone level for each time point for each subject. Imputed scores for each joint/bone were then summed at the time point level together with nonimputed entered scores for the total feature scores for each subject-visit. Imputation for synovitis and osteitis was by baseline observation carried forward to the Week 24 visit. For erosion, JSN, and cartilage loss, the missing score was imputed using the average change of all the other bones/joints in the feature (erosion or cartilage loss) for an individual subject. All statistical analyses were based on two-sided tests and a 5%  *α*-error rate.

Secondary endpoints included absolute change from baseline in RAMRIS erosions, synovitis and osteitis, and CARLOS. Efficacy parameters included change from baseline in TSS, DAS28 (ESR or CRP), CDAI, SDAI, and ACR20/50/70.

The full analysis set (FAS) population included subjects who received at least one dose of SC-TCZ and was used for reporting safety, tolerability, and efficacy. Exploratory analyses were performed for efficacy endpoints and demographics, and subject characteristics were split by two subgroups: SC-TCZ monotherapy [SC-TCZM: SC-TCZ monotherapy or SC-TCZ + cDMARDs excluding MTX] and SC-TCZ combination therapy [SC-TCZC: SC-TCZ + cDMARDs including all MTX regimens]. The differences between the two groups were too small to test.

An ad hoc subanalysis examined the association between osteitis and synovitis at baseline and the progression of erosion on follow-up at the level of individual bones and joints. Imputed values for nonevaluable bones were not used in this subanalysis.

## 3. Results

### 3.1. Subjects

Seventy-five subjects were screened; 23 were screen failures. The most common reason for screen failure was lack of X-ray evidence of erosions (*n* = 11). Fifty-two subjects were enrolled at 10 Australian centres. Five subjects withdrew from study treatment: two due to AEs (neutropenia and increased ALT), two due to physician decision, and one due to subject decision. Fifty-one subjects completed the study follow-up period; one subject was withdrawn by decision of the investigator due to suspected hypersensitivity reaction, which was subsequently downgraded to an injection site reaction.

Demographics and baseline characteristics are presented in Table 1. The SC-TCZM subgroup consisted of four subjects on TCZ alone and 13 on TCZ and DMARD(s) excluding MTX. Subjects in the SC-TCZM subgroup were older and more likely to be female than subjects in the SC-TCZC group. The mean time since diagnosis in the SC-TCZM group was almost double that of the SC-TCZC group.

Baseline TSS score was higher in the SC-TCZM group than the SC-TCZC group. The SC-TCZM group were also less likely to be using corticosteroids and more likely to have previously used anti-TNF therapy.

### 3.2. Radiography

The mean (SD) change from baseline in TSS was 0.46 (1.29) (Table 2). Based on the nonparametric Wilcoxon test, the change was not statistically different from zero. Ninety percent of subjects had no significant (≥SDC) change in their TSS over the 24 weeks of treatment with SC-TCZ (Table 3). Reduction in TSS was not observed.

The SC-TCZM group had a higher mean increase in TSS than the SC-TCZC group did (Table 2). However, the changes were not statistically significant.

### 3.3. MRI

Two of the 52 subjects were excluded from analysis due to lack of adequate-quality images. One subject had no erosion in the target hand/wrist but was enrolled in the study because a requisite erosion was observed in the other hand or one of the two feet on X-ray at screening. In the pooled FAS population, erosion and cartilage loss both increased numerically, and synovitis and osteitis decreased numerically between baseline and Week 24, but only the decrease in synovitis reached statistical significance (*p* = 0.04) (Table 2). At the subject subgroup level, MRI erosion and cartilage loss increased at Week 24 in both the SC-TZCM and SC-TCZC groups, but not in a statistically significant manner. Most subjects had no significant (≥SDC) change in erosion, cartilage loss, synovitis, and osteitis (Table 3).

There were 40 subjects (80%) with at least one bone with erosion that was also adjacent to a synovial compartment with active synovitis, 36 subjects (72%) with at least one bone with erosion and osteitis, and 29 subjects (58%) with at least one bone with all three features.

### 3.4. Disease Activity

There was a statistically significant improvement in DAS28 for the FAS and both subgroups (*p* < 0.05) by Week 2 and through to Week 24 (Table 4). There was no significant difference between the subgroups. At Week 24, 36 (78%) subjects had achieved DAS28 remission (DAS28 < 2.6). Change in EULAR response at Week 24 showed that one subject (3%) was experiencing a loss of response, while 20 subjects (43%) had improved responses and 13 subjects (28%) had no change.

At Week 24 over 50% of subjects in the FAS had achieved an ACR70 response (Table 4). There were no significant differences between the FAS and subgroups.

The mean SJC and TJC decreased from baseline during the study, reaching a maximum change from baseline at Week 24 (Table 4). SDAI and CDAI scores also decreased during the study (Table 4), with mean activity levels indicating severe disease activity at baseline and low disease activity at Week 24. There was no statistically significant difference between the subgroups.

### 3.5. Patient Reported Outcomes

Disease activity decreased during the study as reported by the physician's global assessment of disease activity (VAS) and subject's global assessment of disease (VAS) (Table 4). There was no significant difference between the subgroups.

FACIT-F scores increased, indicating that fatigue reduced during the study (Table 4). The minimally important difference in FACIT-F change scores is 3 to 4 points [13].

Functional ability, measured by the HAQ-DI, increased during the study (Table 4). A reduction of at least 0.22 indicates a clinically significant improvement. The mean (SD) score at Week 24 was 0.59 (0.56), where a score < 0.50 indicates HAQ-DI remission.

### 3.6. Safety and Tolerability of SC-TCZ

Forty-four subjects (85%) received 24 doses of SC-TCZ. Two subjects withdrew due to AEs (neutropenia and increased ALT) and two subjects experienced events leading to reduced dose frequency (leukopenia and neutropenia).

AEs were experienced by 48 subjects (92%). No grade 4 events or deaths occurred. Eight subjects experienced 11 CTCAE grade 3 events: anaemia, abdominal pain, upper abdominal pain, pyelonephritis, ankle fracture, increased ALT, back pain (*n* = 1 each), neutropenia (*n* = 2), and worsening of RA (*n* = 2).

There were three serious AEs: asthma (Grade 2), anaemia (Grade 3), and Weber B ankle fracture (Grade 3). These events were assessed to have no causal relationship with the study drug and the events resolved without sequelae.

Seven subjects experienced AEs of special interest; these were anaemia, increased ALT (*n* = 2), other abnormal liver function test, herpes zoster, infective bursitis, and basal cell carcinoma.

Immunogenicity studies showed no confirmed cases of development of antibodies to TCZ.

The most common AEs occurring in ≥2% of subjects were upper respiratory tract infection (21 events), RA flare (12 events), neutropenia (8 events), diarrhoea (6 events), mouth ulceration (6 events), and arthralgia (5 events).

### 3.7. Subanalysis at the Joint Level

Excluding bones that were not evaluable because of image artifacts, a total of 1245 of 1250 possible bones were examined for effects of bone erosion and osteitis, and 1144 of 1150 possible bones were examined for effects of adjacent synovitis (RAMRIS-synovitis score does not include the two bones comprising the first carpometacarpal joint). Bone erosions, osteitis, and synovitis at baseline were each associated with bone erosion progression (Table 5), with odds ratios (OR) of 4.9 (95% CI: 2.9, 8.2), 3.8 (95% CI: 2.3, 6.2), and 3.1 (95% CI: 1.7, 5.8), respectively. Among bones with erosion at baseline, a larger proportion of those with concurrent osteitis, that is, “active” erosions (OR = 1.6; 95% CI: 0.9, 2.8) but not those with adjacent synovitis (OR = 1.0; 95% CI: 0.5, 2.0), progressed. Screening for erosion originally excluded 6% of subjects from participating in the study. Screening for at least one bone with active erosion would have excluded 28% more. Requiring subjects also to have synovitis adjacent to the bone with active erosion would have raised the screen failure rate to 42%.

## 4. Discussion

During the study, there were small numeric but not significant increases in mean radiographic erosion and JSN scores, and therefore TSS. Significantly (≥SDC) increased TSS occurred in 10% of patients; most patients had no numeric change in TSS during the study.

Disease activity, based on DAS28, SDAI, CDAI, and the patient- and physician-reported outcomes in pain, fatigue, and functional ability, improved during the study, consistent with other studies of tocilizumab [1, 2, 4]. Adverse events seen during this study were also consistent with the expected safety profile of tocilizumab [1, 2, 4].

**Figure 1:**
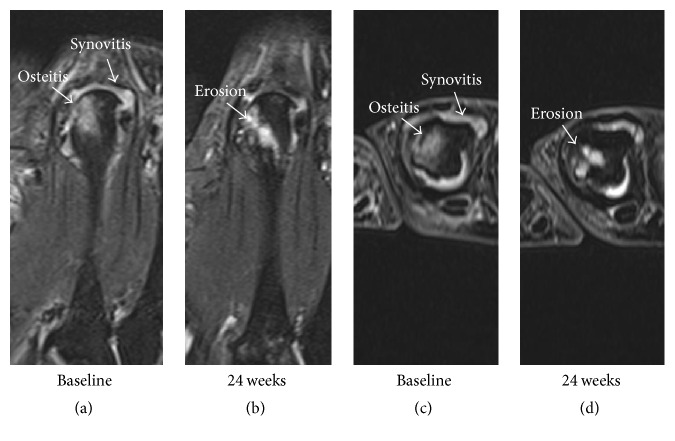
Baseline coronal (a) and axial (c) MR images of the second metacarpophalangeal joint show synovitis and osteitis but no erosion. At 24 weeks (c, d) the synovitis has decreased, and the osteitis has converted to erosion. Cartilage loss had also developed in this joint at 24 weeks (better seen on an adjacent MRI slice not shown). Without an additional time point prior to 24 weeks, however, it is not possible to determine whether the erosion developed prior to the decrease in inflammation or despite it. Further, without a time point after 24 weeks, it is not possible to determine whether the degree of residual synovitis present at 24 weeks is sufficient to drive further progression in joint damage in this case.

Based on MRI, the severity of synovitis and osteitis decreased in the FAS population during the study with statistical significance reached for synovitis. In the exploratory subgroup analysis, the decrease in synovitis was found to be statistically significant in the SC-TCZC subgroup. Lower baseline severity of synovitis in the SC-TCZM group compared to the SC-TCZC group could also have contributed to the difference. Bone erosion and cartilage loss scores were not statistically significantly increased on MRI or radiography in any group at Week 24. Eighteen percent of individual patients did, however, show significant (≥SDC) progression of erosion score. With only two time-points for imaging (screening/baseline and Week 24), it cannot be known whether these structural changes represented progression of disease despite treatment—progression has been reported in other studies, despite clinical remission [14, 15]—or if the structural changes observed at Week 24 actually developed only during the initial period of treatment prior to TCZ reaching its maximum inhibitory effect. Additional time-points would be needed to determine this and whether residual inflammation visible at Week 24 was sufficient to keep joint damage progressing (Figure 1). Another limitation of this study was the small subject numbers in each subgroup, especially SC-TCZM (*n* = 17). Although MRI has been reported to discriminate treatment effects on progression of bone erosion and cartilage loss in placebo-controlled trials with as few as 30 patients per arm and follow-up intervals of only 12 weeks [16–18], more patients and longer intervals than even 24 weeks may be necessary to do so in active-comparator trials, such as this one. Much larger numbers of patients are typically required to demonstrate efficacy in 24 weeks with radiography in placebo-controlled trials, let alone active-comparator trials. The lack of randomisation, and the inability to blind reader to treatment were other limitations. Also, the MRI protocol did not include intravenous gadolinium-based contrast, which may have decreased sensitivity to change for synovitis. Further, the cohort showed lower baseline synovitis and erosion scores by MRI than those reported for some other trials, including ACT-RAY [3], involving patients with longstanding RA.

Results of the ad hoc subanalysis suggest that screening patients not only for the presence of bone erosions but concurrent osteitis and/or adjacent synovitis using MRI could help enrich study populations with patients more likely to progress structurally over short-duration studies.

## 5. Conclusions

A slight decrease in synovitis and osteitis and no significant increase in structural joint damage were observed at Week 24 in the overall population of subjects treated with tocilizumab alone or in combination with methotrexate or other DMARDs. Significant improvement in DAS28 score occurred at each visit during the study. At Week 24, 36 subjects (78%) had achieved DAS28 remission.

Baseline erosions, osteitis, and synovitis each were associated with progression of erosion at the individual bone and joint level. Screening with MRI for the presence of active erosion may enrich RA trial cohorts with likely progressors more effectively than would screening for erosion, osteitis, or synovitis alone.

## Figures and Tables

**Table 1:** Demographics and baseline characteristics.

	FAS (*N* = 52)	SC-TCZM (*N* = 17)	SC-TCZC (*N* = 35)
Female, *n*, (%)	41 (78.9)	15 (88.2)	26 (74.3)
Mean age, years (SD)	56.9 (11.5)	59.2 (12.8)	55.7 (10.9)
Body weight, KG (SD)	78.2 (19.9)	72.9 (16.8)	80.9 (21.1)
Mean duration of RA, years (SD)	7.8 (8.3)	11.2 (8.8)	6.1 (7.6)
Mean SJC28 (SD)	10.7 (6.5)	12.7 (4.9)	9.7 (7.0)
Mean TJC28 (SD)	13.2 (6.2)	12.5 (5.4)	13.5 (6.7)
Mean DAS28-ESR/CRP (SD)	6.4 (1.1)	6.7 (1.0)	6.2 (1.1)
Mean TSS (SD)	16.9 (23.2)	27.4 (32.3)	11.6 (14.9)
Mean X-ray erosion score (SD)	7.4 (9.5)	10.6 (12.3)	5.8 (7.5)
Mean X-ray JSN score (SD)	9.5 (14.4)	16.8 (20.6)	5.9 (8.2)
Mean MRI erosion score (SD)	8.9 (6.7)	9.9 (8.3)	8.4 (5.8)
Mean MRI cartilage loss score (SD)	6.3 (9.5)	9.2 (11.6)	4.8 (7.9)
Mean MRI synovitis score (SD)	4.5 (4.0)	4.3 (5.0)	4.6 (3.5)
Mean MRI osteitis score (SD)	6.6 (10.3)	5.3 (8.1)	7.3 (11.3)
Mean MTX dose, mg/week (SD)	16.9 (6.0)	N/A	16.9 (6.0)
Oral steroid use, *n* (%)	26 (50.0%)	6 (35.3%)	20 (57.1%)
cDMARD use, *n* (%)	44 (84.6%)	10 (58.8%)	34 (97.1%)
Prior use of anti-TNF therapy, *n* (%)	4 (7.7%)	3 (17.7%)	1 (2.9%)

cDMARD: conventional disease-modifying antirheumatic drug, DAS28-ESR: disease activity score (28 joints)–erythrocyte sedimentation rate, FAS: full analysis set, TSS: Genant-modified total Sharp Score, MTX: methotrexate, RA: rheumatoid arthritis, SC-TCZC: subcutaneous tocilizumab combination therapy subgroup (including MTX), SC-TCZM: subcutaneous tocilizumab monotherapy subgroup (including DMARD(s) but excluding MTX), SJC28: swollen joint count (28 joints), TJC: tender joint count (28 joints), and TNF: tumour necrosis factor.

**Table 2:** Changes in TSS and components and RAMRIS feature scores from baseline to Week 24.

	FAS	SC-TCZM	SC-TCZC
*TSS (X-ray)*	*n* = 51	*n* = 17	*n* = 34
Mean (SD)	0.46 (1.29)	0.65 (1.30)	0.36 (1.30)
*p*value^*∗*^	0.83	0.93	0.77
*Erosion (X-ray)*	*n* = 51	*n* = 17	*n* = 34
Mean (SD)	0.14 (0.46)	0.16 (0.40)	0.12 (0.49)
*p*value^*∗*^	0.92	0.99	0.89
*JSN (X-ray)*	*n* = 51	*n* = 17	*n* = 34
Mean (SD)	0.32 (0.94)	0.49 (1.0)	0.24 (0.91)
*p*value^*∗*^	0.89	0.97	0.83
*Erosion (MRI)*	*n* = 50	*n* = 17	*n* = 33
Mean (SD)	0.9 (1.66)	0.79 (2.0)	0.96 (1.5)
*p*value^*∗*^	0.51	0.69	0.55
*Cartilage loss (MRI)*	*n* = 50	*n* = 17	*n* = 33
Mean (SD)	0.12 (0.59)	0.09 (0.90)	0.14 (0.35)
*p*value^*∗*^	0.93	0.97	0.93
*Synovitis (MRI)*	*n* = 50	*n* = 17	*n* = 33
Mean (SD)	−1.7 (2.67)	−1.6 (2.5)	−1.8 (2.8)
*p*value^*∗*^	0.04	0.37	0.04
*Osteitis (MRI)*	*n* = 50	*n* = 17	*n* = 33
Mean (SD)	−4.0 (9.58)	−2.9 (7.9)	−4.6 (10.4)
*p*value^*∗*^	0.21	0.55	0.26

^*∗*^Wilcoxon rank-sum test of the hypothesis of no change from baseline at Week 24. Testing change in each subgroup separately; FAS, full analysis set; TSS, Genant-modified total Sharp Score; SC-TCZC, subcutaneous tocilizumab combination therapy subgroup (including MTX); SC-TCZM, subcutaneous tocilizumab monotherapy subgroup (including DMARD(s) but excluding MTX).

**Table 3:** Proportion of subjects showing radiographic and MRI involvement at baseline and change at Week 24.

	Baseline score > 0 (% subjects)	Smallest detectable change (SDC)	Change > 0 (% subjects)	Change > SDC (% subjects)
Erosion (X-ray)	86%	0.84	20%	8%
JSN (X-ray)	69%	1.16	24%	12%
TSS (X-ray)	88%	1.82	31%	10%
Erosion (MRI)	98%	1.58	56%	18%
Cartilage (MRI)	62%	1.06	18%	4%
Osteitis (MRI)	74%	5.14	20%	0%
Synovitis (MRI)	86%	1.76	12%	2%

**Table 4:** Change from baseline to Week 24 in disease activity measures and subject reported outcomes.

	FAS *N* = 52	SC-TCZM *N* = 17	SC-TCZC *N* = 35
*DAS28-ESR*			
*n*	47	14	33
Mean change (SD)	−4.4 (1.5)	−4.3 (1.1)	−4.4 (1.7)
	*p* < 0.0001^*∗*^	*p* = 0.0001^*∗*^	*p* < 0.0001^*∗*^, *p* = 0.77^*∗∗*^
*ACR20 response at Week 24 *			
*n* (%)	43 (91.5%)	12 (85.7%)	31 (93.9%)
95% CI	79.6% to 97.6%	57.2% to 98.2%	79.8% to 99.2%
			*p* = 0.57^#^
*ACR50 response at Week 24*			
*n* (%)	36 (76.6%)	11 (78.6%)	25 (75.8%)
95% CI	62.0% to 87.7%	49.2% to 95.3%	57.7% to 88.9%
			(*p* = 1.00^#^)
*ACR70 response at Week 24*			
*n* (%)	25 (53.2%)	9 (64.3%)	16 (48.5%)
95% CI	38.1% to 67.9%	35.1% to 87.2%	30.8% to 66.5%
			(*p* = 0.36^#^)
*SDAI*			
*n*	45	12	33
Mean change (SD)	−31.6 (16.5)	−34.1 (14.2)	−30.7 (17.3)
*CDAI*			
*n*	46	13	33
Mean change (SD)	−30.0 (15.1)	−33.6 (13.1)	−28.6 (15.8)
*TJC28*			
*n*	47	14	33
Mean change (SD)	−11.1 (7.6)	−11.4 (7.3)	−10.9 (7.8)
*SJC28*			
*n*	47	14	33
Mean change (SD)	−8.8 (6.0)	−9.5 (6.3)	−8.45 (5.9)
*Physician's global assessment of disease activity (VAS)*			
*n*	46	13	33
Mean change (SD)	−48.8 (21.3)	−55.5 (24.4)	−46.2 (19.8)
*Patient's global assessment of disease (VAS)*			
*n*	47	14	33
Mean change (SD)	−48.6 (25.9)	−52.3 (26.7)	−47.0 (25.8)
*Patient's global assessment of pain (VAS)*			
*n*	47	14	33
Mean change (SD)	−41.9 (27.0)	−47.0 (31.0)	−39.7 (25.3)
*FACIT-F*			
*n*	47	14	33
Mean change (SD)	12.6 (10.5)	15.6 (10.5)	11.4 (10.5)
*HAQ-DI*			
*n*	47	14	33
Mean change (SD)	−0.78 (0.59)	−0.79 (0.70)	−0.77 (0.54)

^*∗*^Sign test used to test the hypothesis of no change from baseline to Week 24; ^*∗∗*^Wilcoxon Rank Sum test used to test the hypothesis of no change from baseline between the subgroups; ^#^Fisher's Exact test used to test the hypothesis of no difference between subgroups at Week 24; ACR20/50/70 20%/50%/70% improvement in the American College of Rheumatology response scores; CDAI, Clinical Disease Activity Index; DAS28-ESR, disease activity score (28 joints)-erythrocyte sedimentation rate; FACIT-F, Functional Assessment of Chronic Illness Therapy-Fatigue; FAS, full analysis set; HAQ-DI, Health Assessment Questionnaire-Disability Index; SC-TCZC, subcutaneous tocilizumab combination therapy subgroup (including MTX); SC-TCZM, subcutaneous tocilizumab monotherapy subgroup (including DMARD(s) but excluding MTX); SDAI, Simplified Disease Activity Index; SJC28, swollen joint count (28 joints); TJC, tender joint count (28 joints); VAS, visual analogue scale; CI, confidence intervals.

**Table 5:** Effect of erosion, osteitis, and synovitis at baseline on subsequent progression of erosion.

MRI feature	Number of bones involved	RAMRIS Erosion Scores Mean (SD)		% erosion progression^*∗*^
		Baseline	24 weeks	
−ERO	813	0	0.02 (0.13)	2.7
+ERO	432	1.02 (0.63)	1.09 (0.67)	12.0
−OST	1017	0.24 (0.50)	0.27 (0.55)	4.1
+OST	228	0.89 (0.77)	0.95 (0.78)	14.0
−SYN	465	0.21 (0.50)	0.22 (0.54)	2.8
+SYN	679	0.47 (0.66)	0.52 (0.70)	8.2
+ERO −OST	251	0.95 (0.57)	1.03 (0.63)	10.0
+ERO +OST	181	1.12 (0.70)	1.17 (0.71)	14.9
+ERO −SYN	87	1.10 (0.59)	1.18 (0.65)	12.6
+ERO +SYN	318	1.01 (0.64)	1.07 (0.67)	12.3

Based on 1245 bones evaluable for ERO or OST and 1144 bones evaluable for adjacent SYN. ^*∗*^Erosion progression defined as ≥0.5; ERO: erosion; OST: osteitis; SYN: synovitis.

## References

[1] Kremer J. M., Blanco R., Brzosko M. (2011). Tocilizumab inhibits structural joint damage in rheumatoid arthritis patients with inadequate responses to methotrexate: Results from the double-blind treatment phase of a randomized placebo-controlled trial of tocilizumab safety and prevention of structural joint damage at one year. *Arthritis & Rheumatology*.

[2] Dougados M., Kissel K., Sheeran T. (2013). Adding tocilizumab or switching to tocilizumab monotherapy in methotrexate inadequate responders: 24-week symptomatic and structural results of a 2-year randomised controlled strategy trial in rheumatoid arthritis (ACT-RAY). *Annals of the Rheumatic Diseases*.

[3] Conaghan P. G., Peterfy C., Olech E. (2014). The effects of tocilizumab on osteitis, synovitis and erosion progression in rheumatoid arthritis: Results from the ACT-RAY MRI substudy. *Annals of the Rheumatic Diseases*.

[4] Burmester G. R., Rubbert-Roth A., Cantagrel A. (2014). A randomised, double-blind, parallel-group study of the safety and efficacy of subcutaneous tocilizumab versus intravenous tocilizumab in combination with traditional disease-modifying antirheumatic drugs in patients with moderate to severe rheumatoid arthritis (SUMMACTA study). *Annals of the Rheumatic Diseases*.

[5] Choy E., Caporali R., Xavier R. (2016). FRI0215Subcutaneous Tocilizumab as Monotherapy or in Combination with A csDMARDs in Patients with Rheumatoid Arthritis – Interim Analysis of A Large Phase IV International Umbrella Study, “Tozura”. *Annals of the Rheumatic Diseases*.

[6] Arnett F. C., Edworthy S. M., Bloch D. A. (1988). The American Rheumatism Association 1987 revised criteria for the classification of rheumatoid arthritis. *Arthritis & Rheumatism*.

[7] Aletaha D., Neogi T., Silman A. J. (2010). 2010 Rheumatoid arthritis classification criteria: an American College of Rheumatology/European League Against Rheumatism collaborative initiative. *Arthritis & Rheumatology*.

[8] Genant H. K. (1983). Methods of assessing radiographic change in rheumatoid arthritis. *American Journal of Medicine*.

[9] Genant H. K., Jiang Y., Peterfy C., Lu Y., et al. (1998). Assessment of rheumatoid arthritis using a modified scoring method on digitized and original radiographs. *Arthritis & Rheumatism*.

[10] Østergaard M., Peterfy C., Conaghan P. (2003). OMERACT rheumatoid arthritis magnetic resonance imaging studies. Core set of MRI acquisitions, joint pathology definitions, and the OMERACT RA-MRI scoring system. *The Journal of Rheumatology*.

[11] Peterfy C. G., DiCarlo J. C., Olech E., Bagnard M.-A., Gabriele A., Gaylis N. (2012). Evaluating joint-space narrowing and cartilage loss in rheumatoid arthritis by using MRI. *Arthritis Research & Therapy*.

[12] Lassere M., Boers M., Van Der Heijde D. (1999). Smallest detectable difference in radiological progression. *The Journal of Rheumatology*.

[13] Cella D., Yount S., Sorensen M., Chartash E., Sengupta N., Grober J. (2005). Validation of the Functional Assessment of Chronic Illness Therapy Fatigue Scale relative to other instrumentation in patients with rheumatoid arthritis. *The Journal of Rheumatology*.

[14] Tamai M., Arima K., Nakashima Y. (2017). Baseline MRI bone erosion predicts the subsequent radiographic progression in early rheumatoid arthritis patients who achieved sustained good clinical response. *Modern Rheumatology*.

[15] Sewerin P., Vordenbaeumen S., Hoyer A. (2017). Silent progression in patients with rheumatoid arthritis: is DAS28 remission an insufficient goal in RA? Results from the German Remission-plus cohort. *BMC Musculoskeletal Disorders*.

[16] Beals C., Baumgartner R., Peterfy C. (2017). Magnetic resonance imaging of the hand and wrist in a randomized, double-blind, multicenter, placebo-controlled trial of infliximab for rheumatoid arthritis: Comparison of dynamic contrast enhanced assessments with semi-quantitative scoring. *PLoS ONE*.

[17] Peterfy C., Genovese M. C., Keystone E. (2012). Magnetic resonance imaging substudy in a phase 2b dose-ranging study of baricitinib, an oral janus kinase 1/janus kinase 2 inhibitor, in combination with traditional disease-modifying antirheumatic drugs in patients with rheumatoid arthritis. *Arthritis Rheum*.

[18] Peterfy C., Strand V., Tian L. (2017). Short-Term changes on MRI predict long-Term changes on radiography in rheumatoid arthritis: An analysis by an OMERACT Task Force of pooled data from four randomised controlled trials. *Annals of the Rheumatic Diseases*.

